# Claw Characteristics of Culled Sows from Three Farrow-to-Finish Greek Farms. Part 2: Mechanical Indices of Hoof Horn and Their Associations with Length Measurements and Lesion Scores

**DOI:** 10.3390/vetsci8090175

**Published:** 2021-08-30

**Authors:** Sofia Chalvatzi, Georgios A. Papadopoulos, Fotios Kroustallas, Mihaela Cernat, Vassilis Skampardonis, Christina Marouda, Vasileia Fotiadou, Vasileios Psychas, Theofilos Poutahidis, Leonidas Leontides, Paschalis Fortomaris

**Affiliations:** 1 Laboratory of Animal Husbandry, Faculty of Veterinary Medicine, School of Health Sciences, Aristotle University of Thessaloniki, 54124 Thessaloniki, Greece; geopaps@vet.auth.gr (G.A.P.); fotios2204@yahoo.gr (F.K.); vafot@vet.auth.gr (V.F.); fortomap@vet.auth.gr (P.F.); 2 Department of Epidemiology, Biostatistics and Economics of Animal Production, School of Veterinary Medicine, University of Thessaly, 43132 Karditsa, Greece; tsernat@uth.gr (M.C.); bskamp@uth.gr (V.S.); leoleont@uth.gr (L.L.); 3 Laboratory of Pathology, Faculty of Veterinary Medicine, School of Health Sciences, Aristotle University of Thessaloniki, 54124 Thessaloniki, Greece; cmarouda@vet.auth.gr (C.M.); psychas@vet.auth.gr (V.P.); teoput@vet.auth.gr (T.P.)

**Keywords:** sow’s claw, hoof horn, Young’s modulus, yield stress, maximum stress

## Abstract

The objective of the present study was to investigate the mechanical indices of hoof horn and their association with length measurements and lesion score. The feet of 185 culled sows from three Greek farms (A: 57 sows; B: 64 sows; C: 64 sows) were used. A slice from the dorsal wall of each claw was used to assess by a three-point bending test the Young’s modulus, yield stress and aximum stress values. The available data from a companion study (part 1) on the length measurements and lesion scores of the claws were used to reveal possible relationships. The Young’s modulus values were significantly higher (*p* < 0.001 or *p* < 0.01 depending on location of claw) in the sows of farm C compared to those in sows of farms A and B and in sows of farm B compared to those in the sows of farm A. Yield and maximum stress values were significantly higher (*p* < 0.05 or *p* < 0.001 depending on the location of the claw) in the sows of farm C compared to those in the sows of farm A and in the sows of farm B compared to those in the sows of farm A. An increase in heel-sole length decreased all mechanical indices. Young’s modulus and yield stress were associated with wall lesion severity while maximum stress with wall and heel lesion severity. Overall, we conclude that mechanical efficiency deteriorates as length and lesion score increases.

## 1. Introduction

The hoof, a distinctive feature of ungulate mammals such as horses, cattle and pigs, is a horny capsule composed of keratin that encloses the distal end of the second phalanx, the distal phalanx, and the navicular bone [1]. The hoof has evolved to fulfil protective and mechanical functions [2]. It provides protection to the underlying tissues from the external environment, serves as a weight-bearing element, supports large compressive loads and acts as a shock absorber [3]. As described in cattle, hoof quality is a complex and multidimensional concept that encompasses a diverse range of morphometric, macroscopic, histological and mechanical aspects [4]. A good quality hoof should combine the desirable characteristics of normal growth, sufficient strength and stiffness and adequate resistance to damage and lesions [5]. 

The shape and geometry of the hoof is closely related to its mechanical behavior [6] and hoof horn lesions can be of mechanical origin [7]. In sows, growth abnormalities and lesions of hooves (also called claws) have been the subjects of extensive research [8,9,10,11,12,13,14,15,16,17,18,19]. However there is a general lack of data in literature on the mechanical properties of a sow’s hoof horn. Recently, Van Riet et al. [18] evaluated the mechanical characteristics of hoof horn in Zn-supplemented sows. As stated by the authors, dietary treatment with Zn had no impact on the mechanical indices of the hoof. Nevertheless, studies exploring the relationship between hoof dimensions, mechanical properties and lesion intensity are missing. Driven by this knowledge gap, we intended to investigate aspects of the sow’s hoof mechanics and its relationship with hoof dimensions and lesion severity. Given the fact that breed differences have been reported for sows and boars regarding certain morphological hoof properties such as hoof length, growth and size dissimilarity [20,21,22,23,24] we decided to evaluate hooves of different genotypes. 

The objectives of the present study were (i) to measure the mechanical properties of the hooves of culled sows from the three genetic lines most frequently used in Greek farms (ii) to describe the association of the mechanical indices with hoof length measurements and lesion severity data obtained in a companion study (part 1) [25].

## 2. Materials and Methods

### 2.1. Participating Farms

This study was part of the “T1EDK -02073- FITSOW’ research project investigating the longevity and welfare of sows in commercial Greek farms (financed by the European Regional Development Fund of the European Union and by Greek national funds). The experimental period lasted from January 2019 up to April 2020. Three Greek farrow-to-finish farms with a sow capacity of 250 (farm A), 350 (farm B) and 370 sows (farm C) were included. All herds complied with the EU directive 2001/88/DC on animal welfare. The sows of each farm belonged to different genetic lines (farm A: PIC; farm B: Danbred; farm C: Topigs). All sows were reared throughout their productive life on comparable rations including the same premix with the same quantity of chelated minerals, in order to minimize any effects on claw characteristics due to differences in mineral supplementation. Sows were housed on fully slatted concrete floors during the gestation period and on fully slatted plastic floors during the farrowing period. Each time the sows’ culling was decided by the farm managers, members of the research team were sent to the collaborating slaughterhouses for sample collection.

### 2.2. Slaughterhouse Sampling Protocol

The Council Directive 93/119/EC on the protection of animals at the time of slaughter or killing was applied for all animals. All four feet of 185 culled sows from three Greek farrow-to-finish herds (farm A: 57 sows; farm B: 64 sows; farm C: 64 sows) were collected. The sow’s parity ranged from 0 to 7 (median 5) in farm A, from 0 to 9 (median 6) in farm B and from 0 to 8 (median 6) in farm C. After slaughtering, the feet and the ear tag of each sow were placed in a sealed plastic bag and transferred, under cooling conditions, to the Laboratory of Pathology in the Faculty of Veterinary Medicine in Aristotle University of Thessaloniki. 

### 2.3. Macroscopic Examination of Claws

All claws of front and rear sows’ feet were macroscopically examined and scored for lesions independently by two of us (V.P., C.M.), as described by Lisgara et al. [12] at five anatomical sites: the heel (HL), the sole (SL) the white line (WL), the wall (WA) and the coronary band (CB). Claw length measurements for all claws and dew claws were performed using digital calipers (Facom 150 mm Digital Caliper 0.01 mm) and included (a) the dorsal hoof length (DL) (along dorsal wall from just below the coronary band to the end of the wall), (b) the diagonal hoof length (AL) (along abaxial wall from the bottom of the wall at the toe to the top of abaxial wall-heel junction), (c) the heel-sole length (SHL), which was the length of the abaxial wall (sole) and bulb (heel) that are in contact with the floor surface along ventral surface from the top of the toe to the caudal end of the heel, and (d) the dewclaw length (DCL) (along dorsal wall from just below the coronary band to the end of the wall).

### 2.4. Examination of Mechanical Horn Characteristics

**Figure 1:**
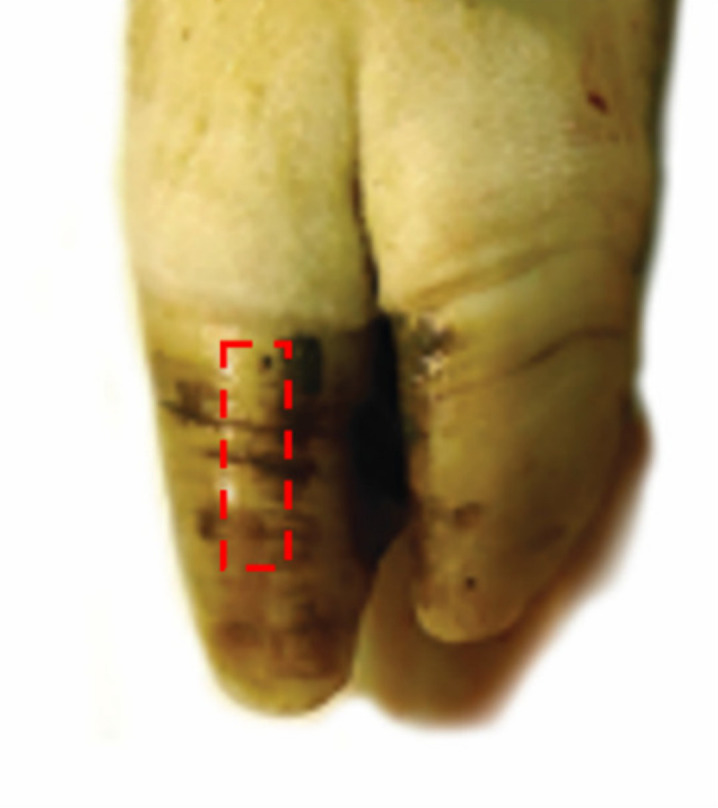
Location of the hoof horn sample.

The examination of the mechanical indices of hoof horn was performed at the Laboratory of Animal Husbandry in the Faculty of Veterinary Medicine in Aristotle University of Thessaloniki. A horn sample from the dorsal wall of each hoof (Figure 1) was cut with a band saw and afterwards the underlying tissues were removed with a scalpel. The mean length, width and thickness of samples, that was measured using a micrometer with an accuracy of 0.01 mm, were 39.43 × 14.00 × 4.60 mm. All samples were put into bags and individually stored at −20 °C. Before analysis, the samples were defrosted for 24 h at 4 °C and weighed. The horn wall samples were subjected to a three-point bending test (Texture Analyzer; Stable Micro Systems Ltd., Surrey, UK) according to the methodology described by Franck et al. [26] and adopted by Van Riet et al. [18]. Due to variations in sample thickness and weights, the test characteristics such as stress area and strain height were adjusted for each sample. The span between the two supports was set to 40 mm and the sample was compressed over a distance of 5.5 mm using a force transducer (load cell, 50 kg) exerted in the middle of the span distance with a loading velocity of 1 mm per minute. The force-deformation curve generated for each sample was transformed to a stress-strain diagram by using the Exponent Software (Stable Micro Systems Ltd., Surrey, UK). Mechanical indices such as Young’s modulus, yield stress, and maximal stress were determined. Young’s modulus is a measure of horn rigidity and stiffness and is represented as the slope of the stress-strain curve in the initial linear. Yield stress is the point on the stress-strain diagram in which the material starts to lose its mechanical function and resistance to further loading due to changes in material properties. Yield stress is represented as the point of the line where the line becomes nonlinear, using a parallel straight line with the same slope as the initial line (strain equal to 1%) and where the intersect with the stress-strain diagram is defined as the yield stress. Maximum stress expresses the maximal load that a sample can withstand [18,26].

### 2.5. Statistical Analysis

All statistical analyses were performed using Stata 13.1 (Stata Statistical Software, College Station, TX, USA) and evaluated at the 5% level of significance. Differences for the measured mechanical indices were compared between farms by one-way ANOVA. Multiple comparisons were done with Tukey’s test. Results on Young’s modulus, yield stress, and maximum stress are presented as mean ± standard deviation (SD).

The possible association between each of the estimated mechanical indices of the hoof sample, namely the Young’s modulus, the yield stress, and the maximal stress, along with the claw length measurements were evaluated in three separate multiple linear regression models. In each of these initial models, the mechanical index was the dependent variable whereas the DL, the AL, and the SHL were independent variables. In addition, a dummy variable, with three categories, coding for the herd of sow’s origin, a dummy variable coding for front or rear foot, a dummy variable coding for right or left foot, a dummy variable coding for lateral or medial hoof, and the sows’ parity were also included as dependent variables. The dummy variable for herd was included to control for unmeasured management factors operating at the herd level (e.g., different genotypes), the dummy variables coding for foot and claw location were included in order to account for the documented different frequencies of claw overgrowth in different feet and toes [8,12,27], and sow’s parity was included to control for the documented effect of the sow’s age on the occurrence and severity of claw overgrowth [12]. In addition to the fixed-effect terms, all the evaluated models included a random-effect term for sow and a random-effect term for foot nested within sow to account for the hierarchical structure of multiple measurements on the same animal and foot. The initially fitted full models were, subsequently, reduced by backwards elimination. Then, we offered previously deleted variables one-by-one to the reduced models, to ensure that no variable with significant impact to the models was omitted. Finally, we created all two-factor interactions between the significant fixed-effect variables in the reduced models and tested their significance by offering them one at a time to the model.

The possible association between each of the estimated mechanical indices of the hoof sample with scores of claw lesions on five anatomical sites of the claw were also evaluated in three separate multiple linear regression models. In each of these initial models the mechanical index was the dependent variable whereas lesion score on the five anatomical sites of the claw, namely the heel (HL), the sole (SL), the white line (WL), the wall (WA) and the coronary band (CB) were independent variables. In addition, a dummy variable, with three categories, coding for herd of sow’s origin, a dummy variable coding for front or rear foot, a dummy variable coding for right or left foot, a dummy variable coding for lateral or medial hoof, and the sows’ parity were also included as dependent variables. In addition to the fixed-effect terms, all the evaluated models included a random-effect term for sow and a random-effect term for foot nested within the sow to account for the hierarchical structure of multiple measurements on the same animal and foot. The initially fitted full models were, subsequently, reduced by backwards elimination. Then, we offered previously deleted variables one-by-one to the reduced models, to ensure that no variable with significant impact to the models was omitted. Finally, we created all two-factor interactions between the significant fixed-effect variables in the reduced models and tested their significance by offering them one at a time to the model.

## 3. Results

### 3.1. Mechanical Indices of Hoof Horn

The mean values (±SD) of Young’s modulus, yield stress and maximum stress of medial and lateral claws of each foot of sows from the three farms are shown in Table 1, Table 2 and Table 3, respectively. In the sows of farm C, the Young’s modulus values were significantly higher compared to those in the sows of farm A for each of the eight claws (*p* < 0.001 for all claws apart from lateral ones in front feet where *p* < 0.010) and compared to those in the sows of farm B for the medial claw of the front left foot and all claws of the rear feet (*p* < 0.001). In the sows of farm B, the Young’s modulus values were significantly higher compared to those in the sows of farm A in all claws except for the lateral claw of the front left foot and the medial claw of rear right foot (*p* < 0.001 with the exception of the lateral claw in the front right foot where *p* = 0.007).

As regards yield stress, the obtained values for the sows of farm C were significantly higher compared to those for the sows of farm A in the medial claw of the front left foot (*p* = 0.024), the medial (*p* = 0.037) and lateral (*p* < 0.001) claws of the rear right foot and the lateral claw of the rear left foot (*p* < 0.001). In the sows of farm B only the yield stress of the lateral claw in front left foot was significantly higher (*p* = 0.017) compared to that of the sows of farm C, while the values for all claws except for medial ones of the front right and rear left feet were significantly higher compared to those in the sows of farm A (*p* <0.05 for front feet and medial claw of rear right foot, and *p* < 0.001 for lateral claws in both rear feet).

The values of maximum stress were significantly higher in the sows of farm C compared to those in the sows of farm A for the lateral (*p* = 0.010) and medial (*p* = 0.020) claws of the front right and left feet, respectively, the medial (*p* = 0.015) and lateral (*p* < 0.001) claws of the rear right foot and the medial (*p* = 0.033) and lateral (*p* <0.001) claws of the rear left foot. No differences were observed between farms B and C. The values of all claws except for the lateral claw of front left foot were significantly higher (*p* < 0.05 for front feet and medial claws of rear feet and *p* <0.001 for lateral claws of rear feet) in the sows of farm B compared to those in sows of farm A.

### 3.2. Lengths and Lesion Scores of Claws

The detailed data on claw length measurements and lesion scores are given in the companion study (part 1) [25].

### 3.3. Associations between Mechanical Indices and Length Measurements

In the reduced final multivariable linear models for the association of Young’s modulus and yield stress index with claw length measurements (Table 4), the farm of the sow’s origin and the SHL retained significance, while no two-way interaction was significant. In the third reduced multivariable linear regression model, the index of maximum stress was associated with the lateral or medial location of the claw and the SHL, while their two-way interaction was not significant. For Young’s modulus a one unit increase in SHL caused an average of 3.14 units decrease (*p* = 0.001, 95% confidence interval (CI): 1.36; 4.92). The Young’s modulus values were significantly higher for the sows of farm C compared to those for the sows of farm A by on average 27.71 units (*p* < 0.001, 95% CI: 14.73; 40.70) and for the sows of farm B compared to those for farm A by on average 17.10 units (*p* = 0.005, 95% CI: 5.04; 29.15). They did not differ (*p* = 0.089) between the sows of farm C and those of farm B.

For yield stress a one unit increase in SHL caused an average of 0.44 units decrease (*p* = 0.006, 95% CI: 0.12; 0.75). The yield stress values did not differ between the sows of farm C compared to those of farm A (*p* = 0.09) or in the sows of farm C compared to those of farm B (*p* = 0.12). However, a statistically significant difference in yield stress values was observed between the sows of farm B compared to the sows of farm A (*p* = 0.001), with the sows of the former having values higher by 2.76 units (95% CI: 1.18; 4.34) on average compared to the sows of the latter farm.

For maximum stress, a one unit increase in SHL was associated with an average 0.71 units (*p* < 0.001, 95% CI: 0.40; 1.03) decrease in the value of maximum stress. The maximum stress score was also on average 0.99 units lower (*p* = 0.039, 95% CI: 0.05; 1.93) on average in medial compared to lateral hooves.

### 3.4. Associations between Mechanical Indices and Lesion Scores

In the three final multivariable linear models (Table 5), the farm of sow’s origin and the claw lesions on the wall of the hoof retained significance, while no two-way interaction was significant. However, for the index of maximum stress, additional associations were detected with heel lesions and the lateral or medial location of the hoof. Young’s modulus was associated with the severity of the wall lesions and the farm of sow’s origin. Particularly, Young’s modulus values were lower by on average 14.21 units (*p* = 0.005, 95% confidence interval (CI): 4.20; 24.22) in hooves with a wall lesion score 2 compared to those with no lesions and lower by on average 11.05 units (*p* = 0.011, 95% CI: 2.52; 19.60) in hooves with wall lesion score 2 compared to those with wall lesion score 1. There was no significant difference (*p* = 0.34) in the Young’s modulus values between hooves without wall lesions and with lesion score 1. Additionally, the Young’s modulus values were significantly higher in sows of farm C compared to those in sows of farm A by on average 33.4 units (*p* < 0.001, 95% CI: 21.00; 45.80), in sows of farm C compared to those in sows of farm B by on average 17.15 units (*p* = 0.004, 95% CI: 5.43; 28.88) and in sows of farm B compared to those in sows of farm A by on average 16.23 units (*p* = 0.012, 95% CI:3.52; 28.94).

Yield stress was also associated with the severity of wall lesions and the farm of sow’s origin (genetic line). Specifically, yield stress values were lower by 2.33 units (*p* = 0.01, 95% CI: 0.54; 4.12) on average, in hooves with a wall lesion score 2 compared to those with no lesions and lower by on average 2.05 units (*p* = 0.008, 95% CI: 0.52; 3.58) in hooves with wall lesion score 2 compared to those with wall lesion score 1. Yield stress values did not differ (*p* = 0.63) between hooves with wall lesion score 1 and hooves without any wall lesion. Concerning the effect of different genetics in yield stress, yield stress values were significantly higher in sows of farm C compared to those in sows of farm A by on average 2.15 units (*p* = 0.01, 95% CI: 0.51; 3.79), in sows of farm B compared to those in sows of farm A by on average 2.49 units (*p* = 0.004, 95% CI: 0.79; 4.20), whereas they did not differ (*p* = 0.65) between sows of farms B and C.

Maximum stress was associated with the severity of wall and heel lesions, the farm of sow’s origin (genetic line) and the lateral or medial location of the hoof. In detail, maximum stress values were lower by 2.90 units (*p* = 0.003, 95% CI: 0.96; 4.83) on average in hooves with wall lesion score 2 compared to those with no lesions and lower by on average 2.28 units (*p* = 0.007, 95% CI: 0.62; 3.94) in hooves with wall lesion score 2 compared to those with wall lesion score 1. Maximum stress values did not differ (*p* = 0.31) between hooves with a wall lesion score 1 and hooves without any wall lesion. Moreover, maximum stress values were lower by 2.32 units (*p* = 0.043, 95% CI: 0.07; 4.56) on average in hooves with heel lesion score 2 compared to those with no heel lesions and lower by on average 1.27 units (*p* = 0.022, 95% CI: 0.18; 2.35) in hooves with heel lesion score 1 compared to those with no heel lesions. However, maximum stress values did not differ (*p* = 0.34) between hooves with heel lesion score 2 and 1. Further, maximum stress values were on average lower by 1.06 units (*p* = 0.026, 95% CI: 0.12; 2.00) in medial hooves compared to lateral ones. Lastly, maximum stress values were significantly higher in sows of farm C compared to those in sows of farm A by on average 2.17 units (*p* = 0.031, 95% CI: 0.20; 4.15), in sows of farm B compared to those in sows of farm A by on average 2.70 units (*p* = 0.01, 95% CI: 0.65; 4.73), whereas they did not differ (*p* = 0.58) between sows of farms B and C.

## 4. Discussion

Hoof horn is a biological composite of keratinized material that is naturally designed to serve as a protective cover for the internal structures and as a weight bearing element [3]. The geometric balance, structural integrity and mechanical efficiency of the hoof capsule are key determinants of its functionality. In sows, there is limited knowledge regarding the sow’s hoof mechanics. Available published data dates back to 1984 when Webb et al. [28] measured the average compressive strength of the hoof wall of 22 pigs. Recently van Riet et al. [18] evaluated with bending tests the Young’s modulus, yield stress and maximum stress in abaxial hoof wall samples of medial and lateral hooves of front right foot of 36 sows. In the present study, we intentionally decided to examine, also with bending tests, the mechanical indices in the dorsal wall samples of all four feet of 185 sows (1480 hoof samples) in an attempt to gain reference values for future studies. Values obtained in this study are higher compared to those reported by van Riet et al. [18] for samples subjected to the same test velocity (1 mm per minute). In line with this finding, Franck et al. [26] stated that in bovines, the modulus of elasticity of dorsal wall horn samples were found to be significantly higher compared to that of abaxial ones.

Our experimental approach revealed evident variations between farms in regard to hoof mechanical characteristics. The companion study (part 1) [25] showed that overgrown hooves are associated with a greater lesion score. This study demonstrated that a decline in mechanical efficiency accompanies hooves with increased heel-sole length as well as hooves with high wall lesion scores.

Of importance was the fact that a significant negative correlation was revealed between Young’s modulus values and hoof wall lesion scores. Young’s modulus is a measure of horn stiffness, or in other word, a measure of the hoof’s ability to resist deformation during floor contact and avoid lesion formation [26]. The hoof wall is expected to withstand high loads without excessive deformation and simultaneously to avoid fracture. This mission can be fulfilled due to its viscoelastic behavior. When it is subjected to high or rapid stress it deforms in an elastic manner, whereas under constant stress deformation occurs slower in a viscous manner. Viscoelastic materials exhibit higher Young’s modulus and strength but a lower breaking strain with increasing strain rate [29].

Findings in this study indicate that lesion development is associated with impairment of mechanical efficiency. The latter could be induced by excessive hoof growth since a statistically significant negative correlation was also detected between heel-sole length and the Young’s modulus values. To our knowledge, this is the first study reporting an association between wall stiffness and lengths and lesion scores in a sow’s hoof. Borderas et al. [30] found a negative correlation between another hoof mechanical property, namely hardness, and the severity of lesions in bovine samples, indicating that softer hoof horn was related to increased severity of lesions.

Although hoof wall is considered a “dead tissue” [29], the stratum medium of the hoof wall is considered one of the most fracture-resistant biological materials known [31]. This middle layer, which is the thickest, is composed of hard keratin and is characterized by a tubular and intertubular structure [32]. The tubules are considered to serve mechanical functions, such as fracture and crack-resistance while the intertubular horn provides mostly stiffness and fracture toughness [33]. Regional differences of tubular density in equine hoof horn was reported by Reilly and al. [34] and led to the conclusion that increased tubule density is necessary for smooth energy transfer and crack avoidance. The existence of distinct zones of morphologically different tubules within the sow’s hoof wall were recently identified by Varagka et al. [15,16]. In the same study [16], a decrease in tubule density accompanied by an increase in tubule size was reported in hooves with severe wall lesions. Thus, it can be speculated that hoof overgrowth could impose changes in wall architecture with concomitant effects on mechanical strength and lesion susceptibility.

## 5. Conclusions

The present study demonstrated that hoof mechanical indices may vary among sows of different genetic lines. Hoof horn stiffness, as measured by Young’s modulus, seemed to decrease as either sole heel length or wall lesion score increased. Further investigation is needed to elucidate the underlying interrelated mechanisms.

## Figures and Tables

**Table 1:** Young’s modulus values (mean ± SD) in MPa of medial and lateral claws of all feet of culled sows from three genetic lines.

		Farms		
	A	B	C	*p*-Value
**Front right foot**				
Medial claw	57.56 ± 34.57 ^b^	89.59 ± 63.43 ^a^	91.24 ± 55.00 ^a^	<0.001
Lateral claw	64.32 ± 31.21 ^b^	85.66 ± 49.06 ^a^	96.30 ± 54.35 ^a^	0.007
**Front left foot**				
Medial claw	59.62 ± 39.00 ^c^	78.49 ± 55.09 ^b^	94.59 ± 49.09 ^a^	<0.001
Lateral claw	68.70 ± 47.29 ^b^	85.80 ± 57.75 ^a,b^	98.75 ± 52.35 ^a^	0.003
**Rear right foot**				
Medial claw	72.11 ± 67.34 ^b^	91.00 ± 71.64 ^b^	110.35 ± 65.61 ^a^	<0.001
Lateral claw	50.67 ± 38.28 ^c^	85.40 ± 85.01 ^b^	98.58 ± 56.99 ^a^	<0.001
**Rear left foot**				
Medial claw	63.53 ± 42.46 ^c^	81.63 ± 45.76 ^b^	109.01 ± 76.02 ^a^	<0.001
Lateral claw	56.44 ± 35.73 ^c^	70.81 ± 39.58 ^b^	98.91 ± 50.14 ^a^	<0.001

^a,b,c^ Mean values within the same row with different superscript letter are significantly different (*p* < 0.001 or *p* < 0.01 depending on the location of the claw). MPa = MegaPascals

**Table 2:** Yield stress values (mean ± SD) in MPa of medial and lateral claws for all feet of culled sows from three genetic lines.

		Farms		
	A	B	C	*p*-Value
**Front right foot**				
Medial claw	13.04 ± 5.21	16.27 ± 7.80	14.54 ± 6.04	0.090
Lateral claw	14.11 ± 4.89 ^b^	17.47± 6.61 ^a^	17.06 ± 6.96 ^a,b^	0.019
**Front left foot**				
Medial claw	13.24 ± 7.25 ^b^	15.85 ± 8.06 ^a^	16.50 ± 7.55 ^a^	0.024
Lateral claw	15.03 ± 7.74 ^b^	16.66 ± 5.12 ^a^	14.13 ± 5.26 ^b^	0.017
**Rear right foot**				
Medial claw	13.78 ± 8.09 ^b^	17.35 ± 10.13 ^a^	19.65 ± 28.58 ^a^	0.037
Lateral claw	12.16 ± 4.95 ^b^	17.20 ± 7.21 ^a^	15.77 ± 8.65 ^a^	<0.001
**Rear left foot**				
Medial claw	13.36 ± 7.46	16.43 ± 8.34	16.64 ± 9.22	0.053
Lateral claw	12.22 ± 5.27 ^b^	16.87 ± 7.60 ^a^	16.22 ± 6.53 ^a^	<0.001

^a,b^ Mean values within the same row with different superscript letter are significantly different (*p* < 0.05 or *p* < 0.001 depending on the location of the claw). MPa = MegaPascals

**Table 3:** Maximum stress values (mean ± SD) in MPa of medial and lateral claws of all feet of culled sows from three genetic lines.

		Farms		
	A	B	C	*p*-Value
**Front right foot**				
Medial claw	17.54 ± 5.82 ^b^	21.56 ± 8.05 ^a^	19.42 ± 6.90 ^a,b^	0.020
Lateral claw	19.30 ± 5.78 ^b^	23.30 ± 6.99 ^a^	22.52 ±8.06 ^a^	0.010
**Front left foot**				
Medial claw	18.20 ± 8.08 ^b^	20.26 ± 7.97 ^a^	21.59 ± 8.99 ^a^	0.020
Lateral claw	20.27 ± 8.05	21.96 ± 6.03	22.29 ± 25.86	0.068
**Rear right foot**				
Medial claw	18.86 ± 8.60 ^b^	23.57 ± 12.51 ^a^	21.46 ± 8.13 ^a^	0.015
Lateral claw	16.26 ± 5.36 ^b^	23.23 ± 10.22 ^a^	21.79 ± 11.41 ^a^	<0.001
**Rear left foot**				
Medial claw	18.33 ± 7.88 ^b^	21.70 ± 8.23 ^a^	22.55 ± 10.99 ^a^	0.033
Lateral claw	16.68 ± 5.80 ^b^	21.90 ± 7.58 ^a^	21.57 ± 9.09 ^a^	<0.001

^a,b^ Mean values within the same row with different superscript letter are significantly different (*p* < 0.05 or *p* < 0.001 depending on the location of the claw). MPa = MegaPascals

**Table 4:** The three final multivariable linear regression models for the association between each of the estimated mechanical indices of the hoof’s dorsal wall horn, the Young’s modulus, the yield stress, and the maximal stress, with the claw length measurements, sow’s genetic line, sow’s parity and foot or claw location in 185 culled sows from three Greek farms. Claw length measurements included: the distance from the dorsal skin-horn junction (periople) to the apex of the toe (DL), the distance from the apex of the toe to the skin-horn junction at the heel (AL) and the length of the abaxial wall (sole) and bulb (heel) that are in contact with the floor surface (SHL). In addition to the fixed-effect terms, all the evaluated models included a random-effect term for sow and a random-effect term for foot nested within sow.

	Young’s Modulus			Yield Stress			Maximum Stress		
	Coef.	*p*-Value	95% CI	Coef.	*p*-Value	95% CI	Coef.	*p*-Value	95% CI
**Constant**	92.63	<0.001	73.14; 112.12	17.74	<0.001	14.44; 21.04	27.14	<0.001	24.14; 30.13
**Farm**									
**2 vs. 1**	17.10	0.005	5.04; 29.15	2.76	0.001	1.18; 4.34	-	-	-
**3 vs. 1**	27.71	<0.001	14.73; 40.70	1.49	0.094	−0.25; 3.25	-	-	-
**3 vs. 2**	10.61	0.089	−1.62; 22.85	−1.26	0.121	−2.86; 0.33	-	-	-
**Medial vs. lateral** **location of claw**	-	-	-	-	-	-	−0.99	0.039	−1.93; −0.05
**SHL**	−3.14	0.001	−4.92; −1.36	−0.44	0.006	−0.75; −0.12	−0.71	<0.001	−1.03; −0.40
**Random-effects terms**									
	Estimate	Std. Err.	95% C.I	Estimate	Std. Err.	95% C.I	Estimate	Std. Err	95% C.I
**Sow**	829.52	118.23	627.34; 1096.86	8.74	2.00	5.57; 13.70	18.60	3.06	13.47; 25.68
**Foot**	37.67	83.35	0.50; 2880.23	9.34 × 10^−18^	1.54 × 10^−17^	3.69 × 10^−19^; 2.36 × 10^−16^	3.85 × 10^−14^	6.16 × 10^−14^	1.67 × 10^−15^; 8.87 × 10^−13^

**Table 5:** The three final multivariable linear regression models for the association between each of the estimated mechanical indices of the hoof’s dorsal wall horn, the Young’s modulus, the yield stress, and the maximal stress, with scores of claw lesions on five anatomical sites of the claw, sow’s genetic line, sow’s parity and foot or claw location in 185 culled sows from three Greek herds. The anatomical sites of the claw examined and scored for lesions were the heel (HL), the sole (SL), the white line (WL), the wall (WA) and the coronary band (CB), with severity scores ranging from 0 to 2, except for the CB which ranged from 0 to 1. In addition to the fixed-effect terms, all the evaluated models included a random-effect term for sow and a random-effect term for foot nested within sow.

	Young’s Modulus			Yield Stress			Maximum Stress		
	Coef.	*p*-Value	95% CI	Coef.	*p*-Value	95% CI	Coef.	*p*-Value	95% CI
**Constant**	68.95	<0.001	58.02; 79.88	14.43	<0.001	12.37; 16.49	20.95	<0.001	18.43; 23.48
**Farm**									
**2 vs. 1**	16.23	0.012	3.52; 28.94	2.49	0.004	0.79; 4.20	2.70	0.010	0.65; 4.73
**3 vs. 1**	33.39	<0.001	20.97; 45.80	2.15	0.010	0.51; 3.79	2.17	0.031	0.20; 4.15
**3 vs. 2**	17.15	0.004	5.43; 28.88	−0.34	0.657	−1.85; 1.16	−0.52	0.583	−2.36; 1.33
**Parity**	-	-	-	-	-	-	-	-	-
**Medial vs. lateral** **location of claw**	-	-	-	-	-	-	−1.06	0.026	−2.00; −0.12
**HL**									
**1 vs. 0**	-	-	-	-	-	-	−1.26	0.022	−2.35; −0.18
**2 vs. 0**	-	-	-	-	-	-	−2.32	0.043	−4.56; −0.07
**2 vs. 1**	-	-	-	-	-	-	−1.05	0.344	−3.23; 1.13
**WA**									
**1 vs. 0**	−3.15	0.34	−9.62; 3.31	−0.28	0.634	−1.43; 0.87	−0.62	0.313	−1.82; −0.58
**2 vs. 0**	−14.21	0.005	−24.22; −4.20	−2.33	0.010	−4.12; −0.54	−2.90	0.003	−4.83; −0.96
**2 vs. 1**	−11.05	0.011	−19.60; −2.52	−2.05	0.008	−3.58; −0.52	−2.28	0.007	−3.94; −0.62
**Random-effects terms**									
	Estimate	Std. Err.	95% C.I	Estimate	Std. Err.	95% C.I	Estimate	Std. Err	95% C.I
**Sow**	851.21	118.33	648.19; 1117.80	8.92	1.95	5.81; 13.69	18.08	2.91	13.20; 24.79
**Foot**	80.61	84.90	10.23; 635.14	1.36 × 10^−17^	2.15 × 10^−17^	6.18 × 10^−19^; 3.01×10^−16^	4.40 × 10^−14^	7.24 × 10^−14^	1.75 × 10^−15^; 1.11 × 10^−12^

## Data Availability

Data are available from corresponding author upon request.
